# Differential Involvement of Hedgehog Signaling in Butterfly Wing and Eyespot Development

**DOI:** 10.1371/journal.pone.0051087

**Published:** 2012-12-05

**Authors:** Xiaoling Tong, Anna Lindemann, Antónia Monteiro

**Affiliations:** 1 Department of Ecology and Evolutionary Biology, Yale University, New Haven, Connecticut, United States of America; 2 State Key Laboratory of Silkworm Genome Biology, Southwest University, Chongqing, China; Oxford Brookes University, United Kingdom

## Abstract

Butterfly eyespots may have evolved from the recruitment of pre-existent gene circuits or regulatory networks into novel locations on the wing. Gene expression data suggests one such circuit, the Hedgehog (Hh) signaling pathway and its target gene *engrailed (en)*, was recruited from a role in patterning the anterior-posterior insect wing axis to a role patterning butterfly eyespots. However, while *Junonia coenia* expresses *hh* and *en* both in the posterior compartment of the wing and in eyespot centers, *Bicyclus anynana* lacks *hh* eyespot-specific expression. This suggests that Hh signaling may not be functioning in eyespot development in either species or that it functions in *J. coenia* but not in *B. anynana*. In order to test these hypotheses, we performed functional tests of Hh signaling in these species. We investigated the effects of Hh protein sequestration during the larval stage on *en* expression levels, and on wing size and eyespot size in adults. Hh sequestration led to significantly reduced *en* expression and to significantly smaller wings and eyespots in both species. But while eyespot size in *B. anynana* was reduced proportionately to wing size, in *J. coenia*, eyespots were reduced disproportionately, indicating an independent role of Hh signaling in eyespot development in *J. coenia*. We conclude that while Hh signaling retains a conserved role in promoting wing growth across nymphalid butterflies, it plays an additional role in eyespot development in some, but not all, lineages of nymphalid butterflies. We discuss our findings in the context of alternative evolutionary scenarios that led to the differential expression of *hh* and other Hh pathway signaling members across nymphalid species.

## Introduction

The field of evolutionary developmental biology has revealed that complex traits that are homologous at the morphological level do not necessarily have the same developmental basis [1]. Vulva development in worms [2], and head development in insects [3]–[5] are two examples where conservation of morphology is not underlaid by conservation of developmental mechanisms. These phenomena offer exciting opportunities for investigating the relationship between morphology and underlying genetic circuitry, and gaining insight into how genes get co-opted, redeployed, and gain and lose functionality in gene regulatory networks underlying the development of complex traits.

Nymphalid butterfly eyespots are complex traits that originated once within the nymphalid butterfly clade, roughly 90 million years ago and are, thus, homologous at the morphological level [6]. At the level of gene expression, however, eyespots from different nymphalid species express a very different complement of genes during their early development [6], [7]. The differential gene expression across lineages appears to originate predominantly via a shared and basal gene co-option event followed by lineage-specific gene expression losses [6].

**Figure 1:**
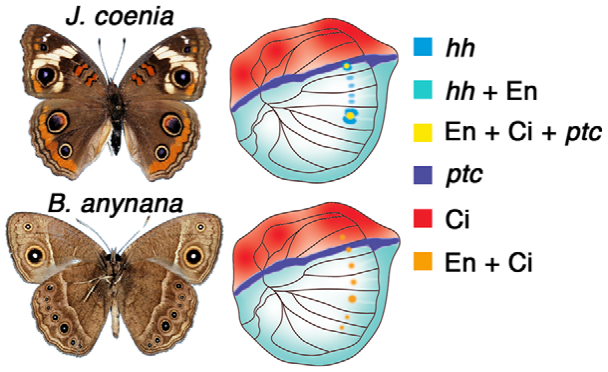
Differential expression of Hh signaling pathway members in *Junonia coenia* and *Bicyclus anynana* larval hindwings. Summary of mRNA (italics) and protein expression data from [8], [9]. *hedgehog* (*hh*) and its receptor *patched* (*ptc*) are expressed in primitive patterns throughout the posterior wing compartment and in a narrow anterior domain abutting that compartment, respectively. These two genes are also expressed in novel domains in *J. coenia* but not in *B. anynana*: flanking the developing eyespot centers, and in the centers, respectively. The other depicted genes share a similar expression pattern between *J. coenia* and *B. anynana*.

*hedgehog* (*hh)* is one of the genes differentially expressed in eyespots across nymphalid species. Transcripts of this gene were originally visualized flanking the center of the future eyespots in *Junonia coenia* larval wings [8] (Fig. 1), but recent stainings in a different nymphalid species, *Bicyclus anynana*, show that *hh* is not expressed in eyespots at comparable larval stages [9] (Fig. 1). The recruitment of *hh* to eyespot development in *J. coenia* was proposed to be part of a larger genetic circuit co-option to the eyespot field [8]. This circuit is the anterior-posterior axis patterning circuit described for fly wings and presumed to play a role in wing patterning and growth across insects [8]. In particular, transcripts of *hh* and its receptor *patched* (*ptc)*, and proteins of the presumptive target gene Engrailed (En) and signal transducer Cubitus interruptus (Ci), are all co-localized to the eyespot centers in *J. coenia* (Fig. 1). These genes share a conserved pattern of expression on the fly and butterfly wing: *hh* mRNA transcripts and En proteins are present in the posterior compartment, Ci protein is present in the anterior compartment, and *ptc* mRNA is present along the anterior-posterior boundary [8], [9] (Fig. 1). It is remarkable then, that while some members of this circuit, such as Ci and En are expressed in *B. anynana* eyespots [8], the Hh receptor *ptc* and *hh* itself, are not [9].

The differential expression of *hh* and *ptc* in *J. coenia* and *B. anynana* eyespots is intriguing and suggests that Hh signaling may not be functional in either species, or may be functional in *J. coenia* but not in *B. anynana* eyespots. In order to test these hypotheses, we provide the first functional tests for the role of Hh signaling in wing and eyespot development in butterflies by directly manipulating Hh function in developing wings of *B. anynana* and *J. coenia.*

**Figure 2:**
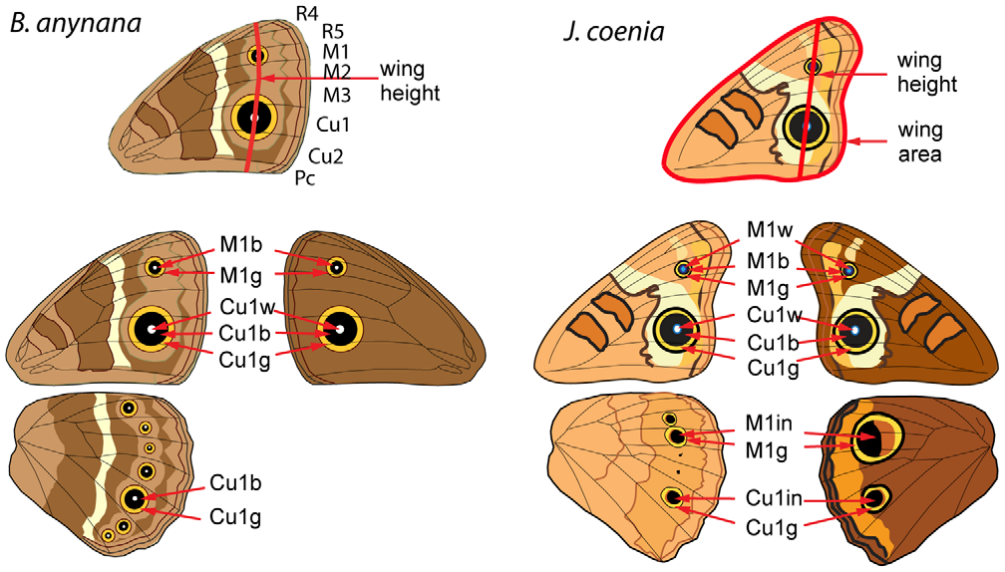
Measurements taken of adult *B. anynana* and *J. coenia* wings. Wing height and wing area (*J. coenia* only) and a series of eyespot trait diameters for the M1 and Cu1 eyespots were measured on both ventral (left) and dorsal surfaces (right). R5, R4, M1, M2, M3, Cu1, Cu1+Pc, and 1A+2A refer to the wing compartments that were individually measured in *B. anynana* wings only. Their combined height defined the *B. anynana* wing height. w: white center; b: black disc; g: gold ring; in: inner ring (from the distal border of the black patch to the proximal border of the orange patch).

Instead of taking an RNAi approach, which is proving challenging in Lepidoptera [10], we investigated an alternative method to disrupting protein function using antibodies [11]–[13]. In order to manipulate Hh signaling we first tested whether the 5E1 antibody, designed to target mammalian Sonic hedgehog, could also be used to target Hh in invertebrates. The 5E1 antibody inhibits Hh signaling by binding directly to the Hh ligand, sequestering it, and thus preventing Hh binding to the receptor Patched (Ptc) [14], [15]. The upstream elements of the Hh signaling pathway, including the binding of Hh to Ptc, are known to be highly conserved between vertebrates and invertebrates [16]. In order to investigate the likelihood that the 5E1 antibody also recognizes insect Hh proteins we first performed epitope sequence comparisons between mammalian, *Drosophila melanogaster*, and butterfly Hh proteins and then tested whether butterfly protein extracts produce the expected number and size of Hh protein fragments known from the conserved autoproteolysis of genes from this family [17] using Western blots. We compared the expected length of Hh protein products with those known from *D. melanogaster*.

**Figure 3:**
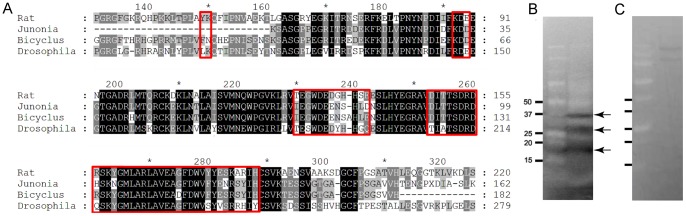
Western blots and similarity of Sonic-Hh and butterfly Hh sequences suggest that 5E1 antibody recognizes Hh in butterflies. (A) Alignment of sequences corresponding to the Sonic-Hh peptide used to make the 5E1 monoclonal antibody [15]. Areas boxed in red correspond to the 5E1 epitope [26], [27]. (B) Western blot with *B. anynana* proteins extracted from wing discs showing three potential Hh fragments with the predicted sizes of 19 kD, 25 kD, and 37 kD (arrows) previously characterized from *D. melanogaster* Hh [21]. (C) No bands were detected with the control NS1 medium. The left lane of each photo is the protein standard.

After confirming the specificity of the 5E1 antibody for Hh nymphalid butterfly proteins we subsequently injected the 5E1 antibody (as well as a vehicle, NS1 control medium) into both *J. coenia* and *B. anynana* larvae at the developmental stage when *hh* transcripts have been detected in eyespots. We monitored levels of a known target of Hh signaling, *en*, in the developing larvae of both species to see if levels of *en* were altered via the antibody injections. After the butterflies pupated and emerged, we measured adult wing and eyespot size. Our experiments support a role for Hh signaling in overall wing growth in both *B. anynana* and *J. coenia* butterflies, but only in eyespot development in *J. coenia.*

**Figure 4:**
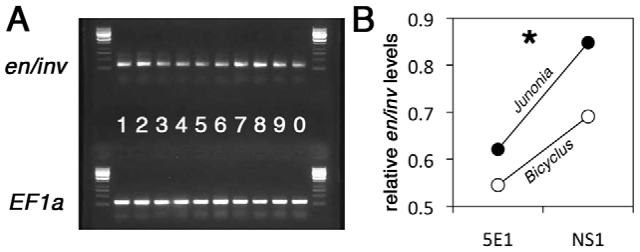
Injections of 5E1 antibody reduce the levels of *en/inv* transcripts one day later in both *B. anynana* and *J. coenia*. (A) PCR amplification of *en/inv* (top) and the house-keeping gene *EF1a* (bottom) from the same samples after injection of either 5E1 antibody or NS1 vehicle. Samples 1–3: *B. anynana* NS1; 4–6: *B. anynana* 5E1; 7–8: *J. coenia* NS1; 9–10: *J. coenia* 5E1. (B) Quantification of brightness levels of *en/inv* PCR amplification relative to brightness levels of the *EF1a* housekeeping gene (averages from data in A). Asterisk (*) indicates a significant difference in *en/inv* relative levels between 5E1 and NS1 injections.

## Materials and Methods

### Sequence alignment and western blots

In order to test antibody specificity, sonic hedgehog protein sequences of rat (SHH-N, Q63673), *D. melanogaster* (AAF56102), *B. anynana* (ADO60878) and *J. coenia* (AAD08931) were aligned using muscle3.6 [18], Clustal X [19] and Genedoc [20]. Sequence identity and similarity were calculated in SIAS (http://imed.med.ucm.es/Tools/sias.html) using the PID_1_ identity method, Blossom 62 matrix, and remainder defaults. Western blots were performed on <40 hr old pupal wing discs of *B. anynana* and band size was compared against blots from 3^rd^ larval wing discs of *D. melanogaster*, with a previously characterized Hh protein profile [21]. In particular, in *D. melanogaster* the full-length form of hedgehog protein (Hh-F) is converted to a species of 39 kD (Hh-U), a signal-cleaved form of Hh-F, which further undergoes autoproteolysis to generate two main products, a 19kD amino-terminal fragment (Hh-N), and a 25 kD carboxyl-terminal fragment (Hh-C). The 25-kD Hh-C species further generates the 16-kD C* species in imaginal disks [21]. Discs were resuspended and homogenized in lysis buffer (50 mM Tris, pH 8.0, 100 mM NaCl, 1% Triton X-100, 10% Glycerol, 1.5 mM EDTA, 1x protease inhibitor cocktail). Homogenates were centrifuged at 14,000 rpm at 4°C for 10 minutes, and the resulting supernatant was collected. A mix of 20 µl supernatant with 5 µl SDS-PAGE loading buffer was separated on a 4%–20% SDS-PAGE gel and transferred to a PVDF membrane (Millipore Corporation cat # K9PN0097). After blocking, the membrane was incubated with the anti-Sonic hedgehog 5E1 antibody (0.14 µg/mL in wash buffer), washed 3 times with wash buffer, 5 min each time, then incubated with goat anti-mouse IgG antibody conjugated to biotin (Invitrogen cat # 643341), washed 3 times with wash buffer, followed by incubation with a Qdot® 625 streptavidin conjugate (Invitrogen cat # 643341). Signals were detected with a standard UV detection system for ethidium bromide-stained gels. A Western blot with NS1 medium, in which the 5E1 antibody is suspended, diluted 1∶500 in wash buffer, was used as control. The monoclonal anti-Sonic hedgehog 5E1 antibody was developed at the Jessell lab at Columbia University [15] and was obtained from the Developmental Studies Hybridoma Bank (DSHB) developed under the auspices of the NICHD and maintained by The University of Iowa, Department of Biological Sciences, Iowa City, IA 52242.

**Figure 5:**
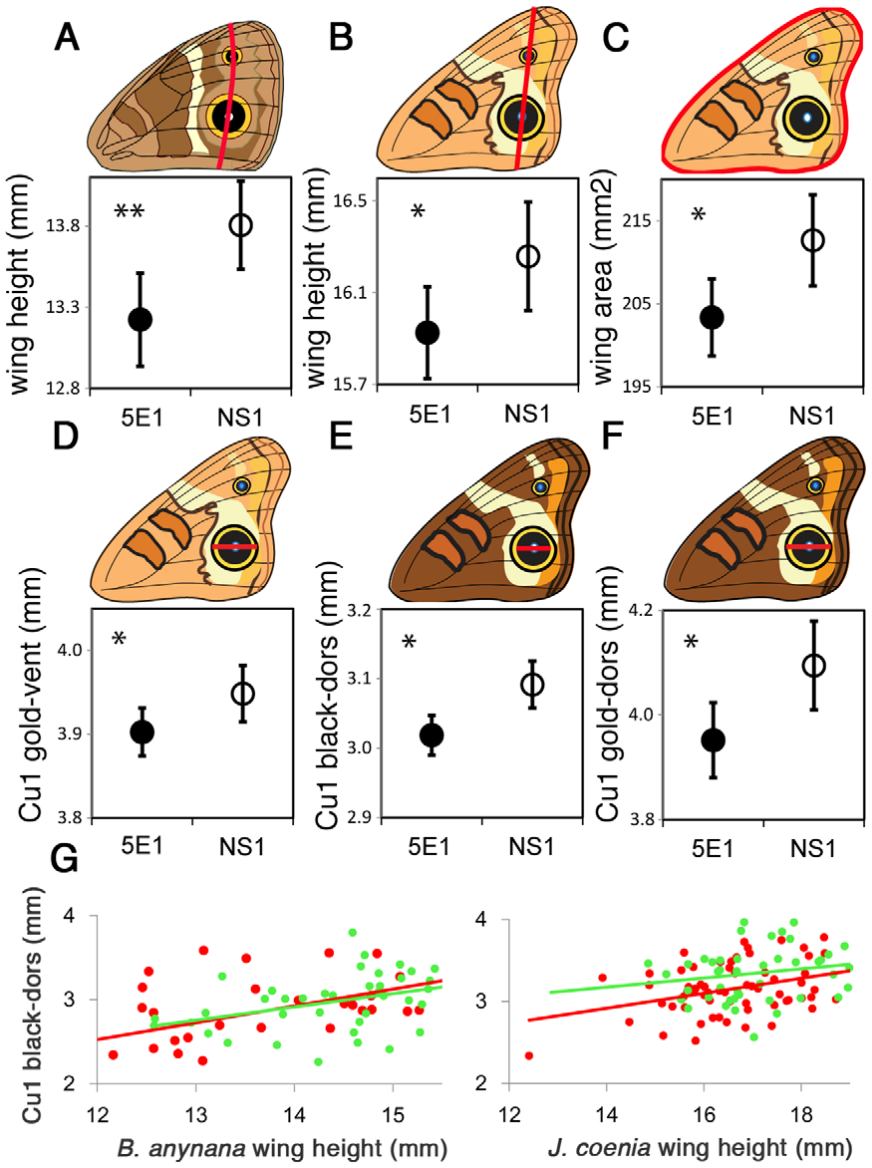
Hh sequestration decreases wing size in both species and relative eyespot size in *J. coenia*. Forewing height in *B. anynana* (A) and *J. coenia* (B) and forewing area in *J. coenia* (C) are smaller in 5E1-injected butterflies compared with NS1-injected controls. Relative eyespot size, e.g., the diameter of the black and gold rings of the Cu1 eyespot on both ventral (vent) and dorsal (dors) surfaces of *J. coenia* is also smaller in 5E1-injected individuals (D-F; mean trait values are displayed for a wing height of 16 mm). GLM analyses use sex as a grouping variable but here sexes are plotted together. (G) Regression of black disc diameter of Cu1 dorsal eyespot on wing height for *B. anynana* (left) and *J. coenia* (right). In *J. coenia*, 5E1-injected individuals (red dots) display significantly smaller Cu1 eyespots relative to NS1-injected individuals (green dots) of comparable size, while *B. anynana* eyespots are not significantly smaller for an individual of a given size.

**Table 1:** F statistics and p-values for GLM analysis testing for differences in wing compartment size in *B. anynana* across treatments.

wing compartment	N (5E1)	N (NS1)	F	p
Forewing R4	35	48	1.40	0.216
Forewing R5	35	48	0.015	0.982
Forewing M1	35	48	1.219	0.231
Forewing M2	35	48	0.158	0.651
Forewing M3	35	48	0.402	0.627
Forewing Cu1	35	48	2.393	0.086
Forewing Cu2+Pc	35	48	0.002	0.957
Forewing 1A +2A	35	48	0.875	0.279

Individuals were injected with either 5E1 antibody or NS1 control media as larvae. Treatment and sex were used as fixed factors and wing height was used as a covariate. Treatment and sex were not significant across analyses.

### Butterfly rearing

*B. anynana* larvae were reared on young corn plants (*Zea mays*) in aluminum mesh cages in a 27°C environmental chamber with a 12∶12 light: dark cycle with a gradual “sunrise” and “sunset,” each one hour in duration. *J. coenia* were reared on narrowleaf plantain (*Plantago lanceolata*) leaves at room temperature (∼25°C), under a natural photoperiod, and inside large plastic containers. After injections, *B. anynana* were reared in mesh sleeve cages with no more than 15 larvae per corn plant. *J. coenia* were reared in the same plastic containers. Butterflies being raised to adults were transferred to hanging net cages after pupation, with no more than 15 pupae per cage, and adults were frozen upon emergence.

**Table 2:** F statistics and p-values for GLM analysis testing for differences in eyespot trait sizes across treatments.

			*B. anynana*				*J. coenia*			
Surface	Wing	Trait	N (5E1)	N (NS1)	F	p	N (5E1)	N (NS1)	F	p
Ventral	Forewing	M1 white	NA	NA	NA	NA	82	59	2.67	0.10365
		M1 black	40	48	2.37	0.09760	82	59	0.003	0.95416
		M1 gold	40	48	0.19	0.82595	82	59	0.68	0.40793
		Cu1 white	40	49	1.32	0.27048	82	59	29.47	<0.0000 ***
		Cu1 black	40	49	3.19	0.04455 *	82	59	0.38	0.53503
		Cu1 gold	40	49	4.59	0.01192 *	82	59	2.36	0.12594
	Hindwing	M1 inner disc	NA	NA	NA	NA	82	59	13.76	0.00026 ***
		M1 gold	NA	NA	NA	NA	82	59	4.75	0.03033 *
		Cu1 black/inner	37	49	1.44	0.23993	82	59	8.97	0.00305 **
		Cu1 gold	37	49	0.98	0.37855	82	59	6.31	0.01271 *
Dorsal	Forewing	M1 white	NA	NA	NA	NA	82	59	23.13	<0.0000 ***
		M1 black	40	46	0.98	0.37734	82	59	13.10	0.00036 ***
		M1 gold	40	47	2.45	0.09086	82	59	12.97	0.00039 ***
		Cu1 white	40	48	1.62	0.20285	82	59	14.98	0.00014 ***
		Cu1 black	37	41	4.37	0.01487 *	82	59	5.07	0.02529 *
		Cu1 gold	37	41	8.37	0.00040 ***	82	59	9.72	0.00206 **
	Hindwing	M1 inner	NA	NA	NA	NA	82	59	0.43	0.50912
		M1 gold	NA	NA	NA	NA	82	59	1.49	0.22313
		Cu1 inner	NA	NA	NA	NA	82	59	3.90	0.04938 *
		Cu1 gold	NA	NA	NA	NA	82	59	7.98	0.00516 **

Individuals were injected with either 5E1 antibody or NS1 control media as larvae. All significant differences correspond to smaller trait sizes in the 5E1-injected individuals. GLM analyses were performed with sex (always significant across traits with females usually displaying a larger trait size than males, not shown); and interaction between line and sex (not significant in all analyses; not shown). *p<0.05, **p<0.01, ***p<0.001.

### Antibody injections

Hh activity was suppressed via injection of the 5E1 antibody into larvae. The 5E1 antibody prevents the Hh signaling ligand from binding to its receptor Patched (Ptc). When this happens, Ptc is able to inhibit Smoothened (Smo) resulting in interactions between Smo, Ci and other protein complexes, which results in the transformation of Ci into a repressor form of Ci (CiR). This repressor form acts as a transcription factor inhibiting the transcription of target genes such as *en* [22]. NS1 medium was used in control injections. Antibody injections were performed using a #701 Hamilton 10 µL syringe with 26–33 gauge needles. Fifth instar larvae were injected on the left side directly posterior to the third thoracic segment with 5 µL of either NS1 medium or 5E1 antibody solutions, at a concentration of 41 µg/mL (in *B. anynana*) and 100 µg/mL (in *J. coenia*). A higher concentration of 5E1 was used for *J. coenia* larvae, in proportion to their higher weight.

**Table 3:** F statistics and p-values for GLM analysis testing for differences in eyespot trait sizes across treatments using wing size as a covariate.

			*B. anynana*				*J. coenia*			
Surface	Wing	Trait	N (5E1)	N (NS1)	F	p	N (5E1)	N (NS1)	F	p
Ventral	Forewing	M1 white	NA	NA	NA	NA	82	59	1.381	0.241
		M1 black	40	48	0.250	0.618	82	59	0.657	0.419
		M1 gold	40	48	0.490	0.486	82	59	1.360	0.245
		Cu1 white	40	49	0.066	0.797	82	59	0.014	0.905
		Cu1 black	40	49	0.115	0.735	82	59	5.455	0.020 *
		Cu1 gold	40	49	0.097	0.757	82	59	4.152	0.031 *
	Hindwing	M1 inner	NA	NA	NA	NA	82	59	1.558	0.213
		M1 gold	NA	NA	NA	NA	82	59	0.001	0.989
		Cu1 black/inner	37	49	0.063	0.803	82	59	1.023	0.313
		Cu1 gold	37	49	0.125	0.725	82	59	1.482	0.225
Dorsal	Forewing	M1 white	NA	NA	NA	NA	82	59	0.418	0.519
		M1 black	40	46	0.049	0.826	82	59	0.612	0.431
		M1 gold	40	47	0.469	0.496	82	59	0.788	0.376
		Cu1 white	40	48	2.961	0.090	82	59	2.123	0.147
		Cu1 black	37	41	0.071	0.790	82	59	5.308	0.022 *
		Cu1 gold	37	41	0.386	0.537	82	59	5.337	0.0218 *
	Hindwing	M1 inner	NA	NA	NA	NA	82	59	0.276	0.600
		M1 gold	NA	NA	NA	NA	82	59	0.259	0.611
		Cu1 inner	NA	NA	NA	NA	82	59	0.094	0.797
		Cu1 gold	NA	NA	NA	NA	82	59	0.318	0.573

Individuals were injected with either 5E1 antibody or NS1 control media as larvae. All significant differences correspond to smaller trait sizes in the 5E1-injected individuals. GLM analyses were performed with sex (always significant across traits with females usually displaying a larger trait size than males; not shown); interaction between line and sex (not significant in all analyses; not shown); and wing size as a covariate. *p<0.05.

### Measurements of *en* transcript levels following injections

We used semi-quantitative PCR to test whether injections of 5E1 antibody had an effect on levels of the putative target gene *en*. We injected nine *B. anynana* and four *J. coenia* larvae with either 5E1 or with NS1, and then we dissected their fore- and hindwings 24 hrs later. We pooled 2 (*J. coenia*) or 3 (*B. anynana*) individuals together before extracting total RNA with a RNeasy Micro kit (Qiagen), and reverse transcribing it with a High-Capacity cDNA Reverse Transcription Kit (Applied biosystems). We used equal amounts of total cDNA in each PCR reaction and primers (Fw: GGA CTG GCC TGC TTG GGT NTA YTG TAC; Rv: TTG AGC CAT CAG TTG CAT AGC NAR NGG RT) that amplified a 313 bp fragment of *en*, within the homeobox region, and that are likely to pick up both *en* and *invected* copies (D. Ramos, pers. comm.). We used *Elongation Factor 1-alpha* (*EF1a*) as a control housekeeping gene. Primers for *EF1a* were Fw: GCY GAR CGY GAR CGT GGT ATY AC and Rv: CAT GTT GTC GCC GTG CCA AC [23]. PCR reactions for each gene were run for 30 cycles. After running the same amount of reaction products on a gel, we quantified the intensity of each PCR band using a digital grayscale image of the gel in Photoshop. We did this by demarcating each gel band inside a constant-size rectangular frame, averaging the intensity of the pixels inside that frame, and collecting the brightness value for the band (100 minus the K-value) using the color picker tool. We corrected the brightness of each band by the correspondent housekeeping gene band brightness by calculating the ratio of the two values. We then used these ratios in a GLM analysis (see below).

### Measurements in adult wings

Left and right adult forewings and hindwings from injected larvae were photographed under a Stereo Discovery V8 Carl Zeiss stereomicroscope equipped with an AxioCam MRC with AxioVision AC Rel. 4.5 software. Pictures were taken using an Anchromat S 0.3X FWD 253 mm lens at 1.0X magnification and saved as TIFF files. Object-Image2.21 [24] and ImageJ 1.42q were used for measurements of the adult wings. In *B. anynana*, we measured the width of each of the wing compartments on the forewing given that Hh*-*inhibition has been known to result in wing compartment specific effects in *D. melanogaster* [25]. Wing cell measurements were taken perpendicular to the wing veins, along the same axis that intersects the two eyespot centers. We used the sum of the wing compartment measurements as a measure of forewing height (Fig. 2). In *J. coenia*, we measured forewing height along the line that crosses the center of both eyespots, and also measured forewing area (Fig. 2). In addition to wing size, we measured the diameter of several eyespot traits on both dorsal and ventral wing surfaces as indicated in Figure 2. All eyespot diameters were taken parallel to the wing veins, along the wing fold.

### Analysis

SPSS Statistics, version 19, was used for statistical analyses of adult wing measurements and for quantification of *en* amplification levels after semi-quantitative PCR. Differences in left and right measurements were examined for each of the treatments, but, given no significant differences between left and right sides, we averaged measurements for the two sides and analyzed the average values thereafter. General Linear Model (GLM) analyses were performed on wing size and eyespot size measurements to test for differences in average trait size between the 5E1- and NS1-injected butterflies using both treatment and sex as fixed variables and a full-factorial design. In order to evaluate eyespot trait size independently of wing size, we performed analyses of covariance on eyespot measurements using forewing height as a covariate. We also used GLM analysis to test for differences in relative levels of *en* amplification in injected animals using treatment (5E1 and NS1) and species (*J. coenia* and *B. anynana*) as fixed factors and a full-factorial design.

## Results

### The 5E1 antibody recognizes butterfly Hedgehog

A comparison between the Hh-N terminal sequence of rat (198 a.a., against which the 5E1 antibody was raised), and the comparable sequence in *B. anynana* (181 a.a.), *J. coenia* (150 a.a.) and *D. melanogaster* (241 a.a.) showed 69%/75%, 70%/68%, and 63%/75% amino acid sequence identity/similarity to rat SHH-N, respectively (Fig. 3A). Recent structural and biophysical analysis revealed that 5E1 binds at the pseudo-active site groove of Shh [26], [27]. We compared the 54 residues forming the 5E1 epitope between rat and each of the three other Hh insect sequences (Fig. 3A, red boxes) and found 72%/80% (*D. melanogaster*), 75%/85% (*B. anynana*) and 68%/ 83% (*J. coenia*) amino acid identity/similarity in these regions.

To confirm that the 5E1 antibody can recognize Hh proteins in insects, proteins from *B. anynana* and from *D. melanogaster* wing discs (where we have knowledge of expected Hh protein size) were extracted and subjected to Western blot analysis. The monoclonal anti-Sonic hedgehog 5E1 antibody, designed to recognize the Shh-N protein fragment in rats [15], detected three bands with the expected sizes in the extracts from *D. melanogaster* (data not shown), and *B. anynana* (Fig. 3B, arrows). No bands were detected with the NS1 control medium in this region (Fig. 3C). The band around 19 kDa is consistent with the size of the Hh-N protein in *D. melanogaster* (Fig. 3B). This indicates that the anti-Shh antibody is probably recognizing the Hh-N protein of both *B. anynana* and *D. melanogaster*. The larger protein, at∼37Kd, is consistent with the Hh-U fragment. Another protein, at ∼25 kD, was also observed in Western blots with proteins extracted from imaginal discs of *D. melanogaster* [21] and could represent an alternative Hh-N size fragment containing the 5E1 epitope. Our data suggest that the monoclonal anti-Sonic hedgehog 5E1 is binding to *B. anynana* Hh, and, by doing so, likely blocking the function of the Hh-N protein as it does for Sonic-Hh.

#### Quantification of *en* expression following injections

Injections of 5E1 significantly reduced the amount of *en/inv* PCR amplicon relative to NS1 injections (F_1,6_ = 8.635; p = 0.026) (Fig. 4). Both *J. coenia* and *B. anynana* responded in a similar way (F_1,6_ = 3.375; p = 0.116), and there was no significant treatment*species interaction (F_1,6_ =  0.409; p = 0.546). The reduction of *en/inv* transcript levels, 24 hrs following the 5E1 antibody injections, suggests that the antibody directly, or indirectly, negatively impacted the transcription of this gene. These experiments, however, do not indicate whether lower *en/inv* transcript levels result from one or both of the *en/inv* expression domains (the posterior compartment or eyespot centers expression domains; see Fig. 1).

### Adult phenotypes resulting from 5E1 antibody and NS1 medium injections

Injections of 5E1 or NS1 medium performed on one side of the larval body led to symmetrical changes in both left and right wings suggesting that the antibody, once injected, circulates throughout the hemolymph and is able to target both sides of the animal, and probably most tissues.

#### Wing size

*B. anynana* and *J. coenia* adults injected with the 5E1 antibody as larvae had a smaller forewing height relative to NS1-injected controls (*B. anynana*: F_1, 82_ = 8.62, p = 0.004; *J. coenia* F_1, 140_ = 4.47, p = 0.036) (Fig. 5A, B). *J. coenia*, where we additionally measured wing area, had a smaller forewing area as well (F_1, 140_ = 6.48, p = 0.012) (Fig. 5C). Wing height reductions in *B. anynana* were due to the compound effect of reductions across all wing compartments as there were no specific compartments that were more affected than others (Table 1). These compartment-specific investigations were not undertaken in *J. coenia,* but the wings appeared also proportionately reduced across the anterior-posterior axis as in *B. anynana*.

#### Absolute eyespot size

5E1 injected butterflies had overall smaller eyespots than NS1 injected butterflies, but while some differences were significant others were not. In *B. anynana* the diameter of the black and gold ring of scales of the largest wing eyespot, the Cu1 forewing eyespot on both dorsal and ventral surfaces, was significantly smaller in 5E1- relative to NS1-injected butterflies (Table 2). In *J. coenia* most eyespots had some trait that was significantly smaller in 5E1-injected individuals relative to controls. This included all eyespot traits measured on the ventral hindwing and dorsal forewing, as well as the white center of the Cu1 eyespot on the ventral forewing, and both measurements for the Cu1 eyespot on the dorsal hindwing (Table 2).

#### Relative eyespot size

The reductions in absolute eyespot size obtained for 5E1-injected butterflies could be due to eyespot differentiation processes having allometrically adjusted to the overall smaller wings. In order to test whether the 5E1 antibody had effects on eyespot size that were independent of its effects on wing size, we performed analyses of co-variance on eyespot traits, corrected for overall wing size (wing height). There was a significant interaction between treatment and wing height for *J. coenia*'s largest eyespot traits, the diameter of the black and gold ring of the Cu1 forewing eyespot on both dorsal and ventral surfaces, but no such interaction in *B. anynana* (Table 3). The converging (non-parallel) regression lines (Fig. 5G) indicate that smaller wings displayed disproportionately smaller eyespots for the 5E1 treatment relative to the NS1 treatment in *J. coenia*, whereas treatment had no apparent effect on eyespot size on larger wings in *J. coenia* (Fig. 5G). This result may simply indicate that stronger effects were seen on smaller animals where the concentration of the antibody was effectively higher (given that the same antibody amount was injected in each animal). In addition, there were significant differences in the relative eyespot size across treatments for *J. coenia* but not for *B. anynana*. 5E1-injected *J. coenia* had significantly smaller eyespots relative to controls for a given wing size (Fig. 5D–F). The wing size data combined with the eyespot size data suggests that Hh signaling promotes wing growth in both butterfly species but promotes larger eyespots only in *J. coenia*. Hh signaling appears to mediate eyespot size in *B. anynana* only indirectly through its effects on general wing growth, but does not appear to be playing a direct role in eyespot development in this species.

## Discussion

We performed functional experiments in butterflies using an antibody that was developed to target Sonic Hh and inhibit this signaling pathway in rats. We showed that this antibody could potentially target other Hh family members, namely Hh from flies and butterflies, because the homologous epitope sequences of all these proteins were quite conserved across species. By performing a Western blot we showed that the antibody targeted protein fragments of the expected size and number as known Hh fragments from *D. melanogaster*, as well as similar sized fragments from *B. anynana* butterflies. Finally, we showed that by injecting the antibody into butterfly larvae, the expression of a known target of Hh signaling in the *D. melanogaster* wing, *en/inv* [28], was affected in *B anynana* and *J. coenia* larval wings. These results collectively indicate that the 5E1 antibody, once injected into butterflies, is likely inhibiting the Hh signaling pathway.

The Hh signaling pathway is involved in cell proliferation in many tissues [29]–[31], and the uncontrolled activation of the Hh signaling pathway has been linked to the growth of tumors in many forms of cancer (reviewed in [32], [33]). The specific role of Hh signaling in wing growth was demonstrated in *D. melanogaster.* Mutants lacking *hh* function had severely reduced wings [25], [34], and reduced *en* expression [28], whereas *hh* gain-of-function mutants had enlarged wings with duplicated posterior compartment vein structures [34]–[36]. The *hh* knock down experiments of Basler and Struhl [34] were performed in *D. melanogaster* during the first larval instar, which is the time interval when the anterior/posterior wing compartments are being established. The comparatively less drastic reduction in wing size resulting from Hh sequestration in *B. anynana* is likely in part the consequence of manipulations late in development, during the fifth instar, after the anterior-posterior axis has already been established, but just as eyespots begin to differentiate. Changes in wing size resulting from Hh sequestration in *B. anynana and J. coenia* during the fifth instar suggest that Hh signaling continues to play a role in wing growth even during later larval development.

While Hh sequestration inhibited wing growth in both butterflies, eyespot trait size reductions independent of wing size were only seen in *J. coenia*. *B. anynana* butterflies displayed small wings with proportionately-sized eyespots, whereas *J. coenia* displayed small wings with disproportionately small eyespots. This indicates that Hh signaling directly affects eyespot development in *J. coenia* but not in *B. anynana,* and that Hh signaling promotes larger eyespots in *J. coenia*.

The functional study done here, directly manipulating Hh availability in *B. anynana* and *J. coenia* butterflies, has illuminated some surprising differences in the effect of Hh signaling on wing development and eyespot development in these two nymphalid butterfly species. Our study shows that *hh* maintains its role in promoting wing growth in butterflies, as it does in *D. melanogaster,* and that *hh* acquired a novel functional role in promoting eyespot development in some butterflies, but not in others. We also note that Hh signaling may have had a more generalized effect on tissue growth, beyond wing growth, which was not documented here.

The presence of Hh signaling in *J. coenia* eyespot development but the absence of such signaling in *B. anynana* requires interpretation from both a mechanistic as well as an evolutionary perspective, i.e., what these differences represent in terms of the proposed recruited circuit and how they could come about in evolution. Originally, the Hh circuit involved in specifying the anterior-posterior wing axis (including *hh*, the Hh receptor *ptc*, the signal transducer *ci*, and the target gene *en*) were proposed to have been co-opted, as a unit, to help build the novel eyespot gene regulatory network [8]. All members of this circuit are present in *J. coenia* butterflies, whereas two of the members are missing in *B. anynana* (*hh* and *ptc*; [9]). In addition, as shown here, disrupting Hh signaling in *B. anynana* does not affect eyespot development. Given these data, it is particularly intriguing that En is being expressed at high levels in the eyespot centers in *B. anynana*, when the gene proposed to activate its transcription (*hh*) is missing. Several explanations for this observation are possible. First, a different member of the Hh family of proteins may activate *en* transcription in *B. anynana*. Presence of additional Hh family members can be tested once the completed *B. anynana* genome becomes available. In arthropods, however, only a single *hh* copy is currently known [37]. Second, *en* transcription in *B. anynana* eyespot centers (and possibly also in *J. coenia*), is being activated by transcription factors unconnected to the Hh signaling pathway. Note that our semi-quantitative PCR experiment cannot distinguish which domains of *en/inv* expression on the wing were actually targeted by the 5E1 antibody injections. It is likely that the lower levels of *en/inv* expression observed following Hh signal inhibition result primarily from the response of cells localized in the posterior compartment of the wing in both species, because this domain is much larger and is also the domain known to be under the control of Hh signaling in *D. melanogaster* wings [28]. If *en/inv* transcription in eyespots is being activated by transcription factors unconnected to the Hh signaling pathway, then either the gene circuit co-opted for eyespot development is different from the one proposed by Keys et al. [8], or the co-opted circuit replaced some of its members in the *B. anynana* lineage but not in the lineage leading to *J. coenia*.

A broader phylogenetic sampling of multiple species for presence and absence of *hh* and *ptc* expression is required to clarify when these genes became associated with eyespots during evolutionary history and to elucidate how and when differential *hh* expression emerged between *B. anynana* and *J. coenia*. Recent comparative gene expression data across 21 nymphalid species and two outgroups showed that the origin of expression of four genes in the eyespot centers (including *en*) happened in a very basal branch of the nymphalid tree, concurrently with the origin of eyespots [6]. Subsequently, many of these gene expression patterns were lost from eyespots in a lineage-specific fashion without loss of eyespots. We proposed that this pattern of rapid, perhaps simultaneous, gene expression gains in association with eyespots, could indicate a gene network co-option event that was followed by the elimination of genes that did not play a role in the development of the novel trait [6]. The same could apply to members of the Hh signaling pathway. All members being co-opted at the same time, as part of a larger network, and some members, such as *hh* and *ptc*, being lost in the lineage leading to *B. anynana*. This gene loss would imply that Hh signaling was not critical for eyespot development in the early nymphalid ancestors. The retention of the whole pathway in *J. coenia* could result from the pathway having been secondarily co-opted to function in eyespot development later in this lineage. An alternative scenario to the single origin of multiple eyespot-associated genes via gene network co-option is a more gradual process of eyespot network modification via lineage-specific additions. Under this scenario, *hh* and *ptc* are co-opted to the *J. coenia* lineage allowing Hh signaling to become functional in this lineage but not in *B. anynana*. Comparative work showed that late additions to the cluster of genes associated with eyespot origins are possible as the gene *Antennapedia* was co-opted into the eyespot centers late and independently in two nymphalid lineages [6], [7]. Only future comparative work involving several more species, however, will determine how exactly *hh* and *ptc* expression in butterfly eyespots evolved.

In conclusion, this work documents an example of a conserved wing pattern, the eyespot, with a single origin within nymphalid butterflies [6] that displays a different developmental basis in different lineages. In one lineage Hh signaling influences adult eyespot size, whereas in another lineage it does not. This example adds to others in the evo-devo literature [2]–[5], [38], where different genes and developmental mechanisms pattern homologous traits.
